# Modulation of the Corticomotor Excitability by Repetitive Peripheral Magnetic Stimulation on the Median Nerve in Healthy Subjects

**DOI:** 10.3389/fncir.2021.616084

**Published:** 2021-03-18

**Authors:** Yanbing Jia, Xiaoyan Liu, Jing Wei, Duo Li, Chun Wang, Xueqiang Wang, Hao Liu

**Affiliations:** ^1^Neuro-Rehabilitation Center, JORU Rehabilitation Hospital, Yixing, China; ^2^Department of Sport Rehabilitation, Shanghai University of Sport, Shanghai, China; ^3^Department of Rehabilitation, JORU Rehabilitation Hospital, Yixing, China

**Keywords:** repetitive peripheral nerve magnetic stimulation, corticomotor excitability, transcranial magnetic stimulation, motor function, brain plasticity

## Abstract

**Objective:** We aimed to examine the effects of repetitive peripheral nerve magnetic stimulation (rPNMS) on the excitability of the contralateral motor cortex and motor function of the upper limb in healthy subjects.

**Methods:** Forty-six healthy subjects were randomly assigned to either a repetitive peripheral nerve magnetic stimulation group (*n* = 23) or a sham group (*n* = 23). The repetitive peripheral nerve magnetic stimulation group received stimulation using magnetic pulses at 20 Hz, which were applied on the median nerve of the non-dominant hand, whereas the sham group underwent the same protocol without the stimulation output. The primary outcome was contralateral transcranial magnetic stimulation (TMS)-induced corticomotor excitability for the abductor pollicis brevis of the stimulated hand in terms of resting motor threshold (rMT), the slope of recruitment curve, and peak amplitude of motor evoked potential (MEP), which were measured at baseline and immediately after each session. The secondary outcomes were motor hand function including dexterity and grip strength of the non-dominant hand assessed at baseline, immediately after stimulation, and 24 h post-stimulation.

**Results:** Compared with the sham stimulation, repetitive peripheral nerve magnetic stimulation increased the peak motor evoked potential amplitude immediately after the intervention. The repetitive peripheral nerve magnetic stimulation also increased the slope of the recruitment curve immediately after intervention and enhanced hand dexterity after 24 h. However, the between-group difference for the changes was not significant. The significant changes in hand dexterity and peak amplitude of motor evoked potential after repetitive peripheral nerve magnetic stimulation were associated with their baseline value.

**Conclusions:** Repetitive peripheral nerve magnetic stimulation may modulate the corticomotor excitability together with a possible lasting improvement in hand dexterity, indicating that it might be helpful for clinical rehabilitation.

## Introduction

Repetitive peripheral magnetic stimulation (rPMS) is a safe, non-invasive treatment method for motor impairment and pain in people with neural or musculoskeletal disorders because it can penetrate deeper structures with painless stimulation and can produce muscle contractions and sensory afferents (Beaulieu and Schneider, 2015). With the coil (pulse generator) applied to the muscles, previous studies have demonstrated that rPMS can reduce spasticity and improve motor control of paretic limbs in individuals with stroke (Struppler et al., 2003, 2007; Beaulieu et al., 2015). The underlying mechanism of such clinical improvement is associated with cortical plastic effects. For instance, using neuroimaging tools and transcranial magnetic stimulation (TMS) in stroke, researchers have shown that rPMS on paretic limb muscle can induce the activation of the frontoparietal loops (Struppler et al., 2007; Gallasch et al., 2015) and increase corticomotor excitability (Gallasch et al., 2015; Beaulieu et al., 2017) in the lesioned hemisphere. Such neurophysiological changes can explain the improvement of motor function after rPMS.

Transcutaneous electrical stimulation to the peripheral nerves (PNS) is a common intervention used to treat motor impairment for clinical rehabilitation. In humans, evidence suggests that PNS enhances the excitability of the motor cortex. In our previous study, we applied PNS to the radial and ulnar nerves in the paretic upper limb and showed that PNS for 1 h increased the corticomotor excitability, which was assessed by TMS in both hemispheres, and improved the dexterity performance of the affected upper limb in people with chronic stroke (Liu and Au-Yeung, 2017). When the stimulation is performed over the median nerve, PNS upregulated cortical excitability in both healthy subjects and patients with central nervous system lesions (Farias da Guarda and Conforto, 2014; Chen et al., 2015). To compare with PNS, stimulating the peripheral nerves with magnetic pulses, in a process called repetitive peripheral nerve magnetic stimulation (rPNMS), preferentially activates the lower motor nerves with minimal activation of cutaneous fibers so that is considered as a painless method (Szecsi et al., 2014; Beaulieu and Schneider, 2015). Furthermore, rPNMS does not need skin preparation and the patient can remain clothed. These advantages of magnetic stimulation might allow rPNMS to be used more widely in clinical practice. In terms of clinical effects, few studies demonstrated rPNMS could reduce the muscle spasticity in children with cerebral palsy (Flamand et al., 2012) and improve motor function in healthy people (Kremenic et al., 2004). However, whether rPNMS can induce modulatory effects within the motor cortex is not known. The aim of the present study was therefore to investigate if rPNMS can induce corticomotor excitability changes in normal subjects. We hypothesized that one session of rPNMS to the arm could enhance corticomotor excitability in the contralateral hemisphere together with motor function improvement of the ipsilateral upper limb. Understanding the corticomotor effects of rPNMS in healthy subjects might aid in the use of rPNMS as an evidence-based treatment for clinical rehabilitation.

## Materials and Methods

### Subjects and Study Design

Forty-six young physiotherapy interns in JORU Rehabilitation Hospital were recruited in the study after providing written informed consent. This sample size was based on the data from our pilot study by assuming a type I error of 5% and power of 80%. All subjects were randomly assigned to either an rPNMS group (*n* = 23, 14 males, nine females; age = 21.17 ± 1.27 years; right-handed, 21 subjects) or a sham group (*n* = 23, 12 males, 11 females; age = 21.30 ± 1.22 years; right-handed, 21 subjects) according to a coded lot picked by them. The inclusion criteria were normal physical status, uneventful past and present medical conditions on the non-dominant upper extremity. Subjects were excluded if they had a history of musculoskeletal or neurological pathology affecting the non-dominant upper limb, signs of cognitive impairments, or contraindications for TMS including a history of epilepsy and presence of metal in the head region or a cardiac pacemaker (Rossi et al., 2009).

**Figure 1:**
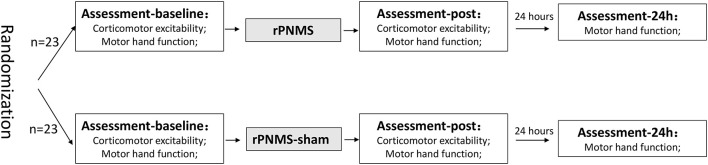
Flowchart of the experimental procedure.

All subjects underwent one session of intervention according to the protocol for the specific group (rPNMS or sham). TMS-induced corticomotor excitability and motor function of the non-dominant hand were evaluated at baseline and immediately after the intervention. To examine the lasting effects, motor hand function was also assessed 24 h after the intervention. The assessment and intervention were delivered by specific but different physical therapists to realize the allocation concealment. Figure 1 presents the experimental procedure for this study. The study protocol was approved by the Research Ethics Committee of the JORU Rehabilitation Hospital (No.: 20190702A01) and was conducted as per the Declaration of Helsinki (World Medical Association, 2013).

### Application of Magnetic Stimulation: rPNMS and rPNMS-Sham

Subjects were seated, and their non-dominant forearm, in a supine position, was placed on a pillow at rest on the table in front of them. Magnetic stimulation was applied on the median nerve of the non-dominant hand over the volar side of the forearm at 3–4 cm apart from the distal wrist crease using the Magneuro100 stimulator (VISHEE Company Limited, Nanjing, China) and a figure-of-eight coil (outer diameter of each wing: 80 mm). The coil was positioned such that the handle was perpendicular to the arm (Gallasch et al., 2015).

The stimulation consisted of 60 trains with a pulse frequency of 20 Hz at a train duration of 2 s and an inter-train interval of 8 s. Thus, a total of 2,400 pulses were delivered in the whole session over 10 min. This specific protocol selected in the current study was based on previous literature that reported the longer lasting effects of motor control with 20 Hz peripheral magnetic stimulation and 2,000–4,000 stimuli with OFF/ON ratio at the vicinity of 4 was most used for sensorimotor impairments (Gallasch et al., 2015). The intensity of stimulation was set at 150% threshold intensity which was defined as the lowest output intensity for inducing visible contractions, such as thenar apposition and flexion of the index and middle fingers with a single magnetic pulse on the median nerve (Gallasch et al., 2015). The mean threshold intensity was 10.35 ± 2.25% of the maximum stimulator output for the rPNMS group and 10.65 ± 2.03% for the sham group. Therefore, the applied mean stimulation intensity was 15.75 ± 3.45% for the rPNMS group and 16.25 ± 3.01% for the sham group. For the sham group, the reverse side of the coil contacted the arm so that no magnetic output was given to the target median nerve with the same noise generated from the stimulator as the rPNMS group.

### Outcome Measures

#### Corticomotor Excitability

Changes in corticomotor excitability for the abductor pollicis brevis muscle (APB) of the non-dominant hand were assessed using the Magneuro 100 stimulator connected with the matching motor evoked potential (MEP) detection module (bandpass: 20–500 Hz) and figure-of-eight coil (VISHEE Company Limited, Nanjing, China). The EMG signals were captured by a pair of self-adhesive surface electrodes placed over the tendon and belly of the APB muscle, with the ground electrode placed over the ulnar styloid process of the arm. The MEP detection module then recorded and processed the signals, and MEP data were output on the computer screen. The corticomotor excitability was evaluated using three parameters: resting motor threshold (rMT), the slope of the MEP recruitment curve (RC slope), and the peak amplitude of MEP (peak MEP). All three parameters have been shown to have good test-retest reliability (intraclass correlation coefficient ≥ 0.75) in our previous study (Liu and Au-Yeung, 2014).

During the TMS assessment, subjects sat on a high-back chair with their arms, legs, neck, and back supported. The examiner placed the coil tangentially on the scalp over the hand representation area of the primary motor cortex (M1) contralateral to the non-dominant hand, with the coil handle pointed backward and at 45° from the midline sagittal plane of the skull. A single magnetic pulse was generated for assessment. The optimal site which is called a “hotspot” was located such that it consistently elicited the largest MEP with the lowest TMS intensity by moving the TMS coil in 1 cm steps over the M1 contralateral to the target APB with a TMS intensity above 60% of the maximum output (Liu and Au-Yeung, 2014). After the hotspot was identified, the rMT was defined as the lowest TMS intensity which could produce MEP amplitudes of at least 50 μV for the relaxed APB muscle in at least 5 out of 10 consecutive TMS stimuli (Darling et al., 2006). Afterward, the MEPs were recorded at stimulation intensities at 1.0, 1.1, 1.2, 1.3, 1.4, and 1.5 of rMT for every five stimuli (Liu and Au-Yeung, 2014). After a resting period of 1 min, the same procedure was repeated. Therefore, the RC in the present study was plotted with the average MEP amplitude of 10 stimuli against the corresponding TMS intensities from 1.0 to 1.5 rMT. With rMT above 72% maximum stimulation output, RC of two subjects were plotted using the intensity from 1.0 to 1.3 of rMT. The RC slope was calculated as the linear slope of this stimulus-response curve. The peak MEP amplitude was identified as the maximum mean MEP evoked by the TMS stimuli in examining the recruiting curve.

#### Motor Hand Function

##### Grip Strength

The maximal grip strength of the non-dominant hand was measured using the Jamar dynamometer (Sammons Preston, Rolyon, Bolingbrook, IL, USA) while the subjects were sitting, with the elbow kept at 90° flexion and the forearm in neutral pronation. Three trials of maximal grip force were recorded, and the mean value was calculated.

##### Hand Dexterity

The dexterity of the non-dominant upper extremity was evaluated using the Purdue pegboard. During the assessment, subjects were required to pick up small pins using the non-dominant hand and insert the pins into holes of the board along the column ipsilateral to the tested hand consecutively. The hand dexterity score was the mean value of the number of pins inserted into the holes in 30 s for three trials.

### Data Analysis

The SPSS statistical software package (Version 20.0) was used for data analysis. The demographic characteristics of the subjects and all outcome measures are represented by the calculations of means and standard deviations (SDs). Assumption of normality for all outcomes data was validated using the Shapiro–Wilk test. An independent sample *t*-test was conducted to examine the differences in baseline measurements between the two groups. The corticomotor excitability in terms of rMT, RC slope, and peak MEP amplitude and motor hand function outcomes of grip strength and dexterity function were examined with two-way repeated-measures analysis of variance (ANOVA) with within-subject factor for time (two levels on corticomotor excitability: baseline, post-stimulation; three levels on motor hand function: baseline, post-stimulation, 24 h afterward) and between-subject factor for the groups (rPNMS and sham). In case of significant or potentially significant time*group interaction effect, *post hoc* comparisons were performed using one-way repeated measures ANOVAs for each group with factor time (two levels on corticomotor excitability: baseline, post-stimulation; three levels on motor hand function: baseline, post-stimulation, 24 h afterward) with the Bonferroni correction. The significance threshold was set at 0.05.

For any significant changes in each outcome measure after rPNMS, the Pearson correlation coefficient was used to examine its relationship with its baseline value. The level of significance was set at 0.05.

## Results

Valid data were obtained for all 46 participants of the two groups. None of the subjects reported any feeling of pain or discomfort during either rPNMS or sham stimulation. The demographic characteristics of the subjects in the two groups were comparable (Table 1).

**Table 1:** Demographic characteristics of the subjects assigned into two groups.

	rPNMS (*n* = 23)	Sham (*n* = 23)
Age (years), mean ± SD	21.17 ± 1.27	21.30 ± 1.22
Male (*n*)	14	12
Female (*n*)	9	11
Right-handed (*n*)	21	21
Left-handed (*n*)	2	2

**Figure 2:**
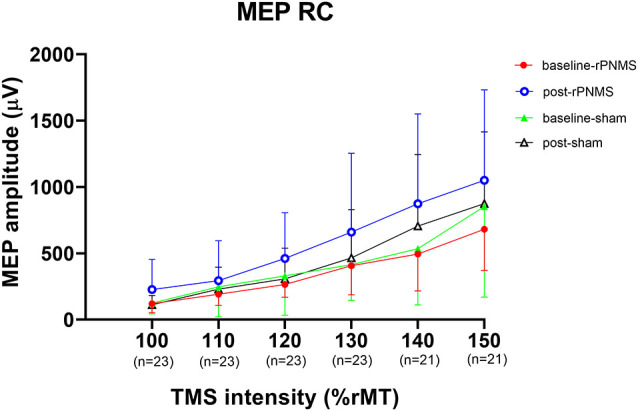
The motor evoked potential (MEP) recruitment curves plotted with MEP amplitude (μV) against the intensity of the transcranial magnetic stimulation (TMS) stimulus (%rMT) for two groups at baseline and post-stimulation. The solid dots (red) and open circles (blue) represent the MEP values for repetitive peripheral nerve magnetic stimulation (rPNMS) at baseline and post-stimulation respectively; the solid (green) and open (black) triangles were for that of sham stimulation at baseline and post-stimulation, respectively.

**Figure 3:**
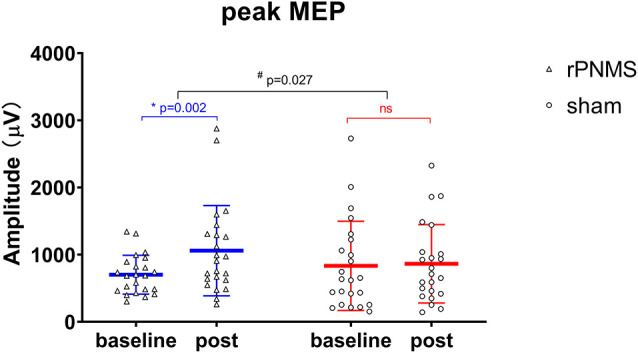
The peak MEP amplitude (μV) was assessed at baseline and immediately after intervention: rPNMS (blue line) and sham (red line). The open triangles and circles represent the individual data in the rPNMS and sham group respectively. The change after rPNMS was different from that after sham stimulation (two-way repeated-measures ANOVA, time*group interaction effect; ^#^*p* = 0.027). *Post hoc* analysis showed significant improvement in peak MEP amplitude after rPNMS (*p = 0.002) but not sham stimulation. ns, not significant.

The MEP recruitment curves plotted with MEP amplitude against the intensity of the TMS stimulus for the two groups were shown in Figure 2. The statistical analysis of all outcomes at baseline with an independent *t*-test showed that there were no significant differences between the two groups (*p* > 0.05). rPNMS resulted in a significant increase in peak MEP amplitude than the sham stimulation. Two-way repeated-measures ANOVA revealed a significant effect of time (*F* = 7.458, *p* = 0.009) and a significant effect of time *group interaction (*F* = 5.261, *p* = 0.027) with no significant effect of group (*F* = 0.043, *p* = 0.838). *Post hoc* comparison showed a significant difference in peak MEP amplitude in the rPNMS group (*p* = 0.002) but not in the sham group (*p* = 0.762) at post-stimulation compared with baseline (Figure 3).

**Figure 4:**
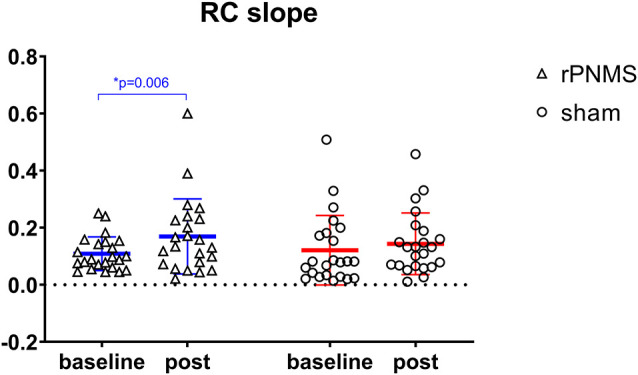
The slope of the MEP recruit curve was assessed at baseline and immediately after intervention: rPNMS (blue line) and sham (red line). The open triangles and circles represent the individual data in the rPNMS and sham group respectively. There was no significant difference in RC slope change between the two groups. The significant change after rPNMS is presented as *p = 0.006.

**Figure 5:**
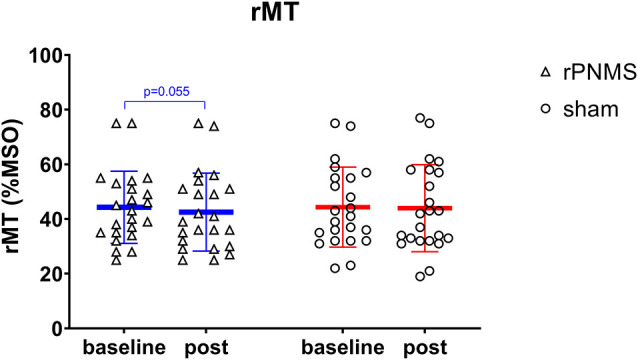
The rMT (%MSO) was assessed at baseline and immediately after intervention: rPNMS (blue line) and sham (red line). The open triangles and circles represent the individual data in the rPNMS and sham group respectively. There was no significant difference in rMT change between the two groups. The change after rPNMS was showed a trend toward reduction as *p* = 0.055.

rMT and RC slope were respectively decreased and increased in all participants over time: two-way repeated-measures ANOVA demonstrated a significant effect of time (*F* = 4.085, *p* = 0.049 for rMT; *F* = 8.205, *p* = 0.006 for RC slope) but no significant effects of group (*F* = 0.030, *p* = 0.863 for rMT; *F* = 0.056, *p* = 0.814 for RC slope) or group * time interaction (*F* = 1.673, *p* = 0.203 for rMT; *F* = 1.735, *p* = 0.195 for RC slope). *Post hoc* comparison revealed a significant increase in the RC slope (*p* = 0.006; Figure 4), whereas the change in rMT was not significant but showed a trend toward reduction (*p* = 0.055) after rPNMS (Figure 5). Both rMT and RC slope for corticomotor excitability remained unaltered after sham stimulation.

**Figure 6:**
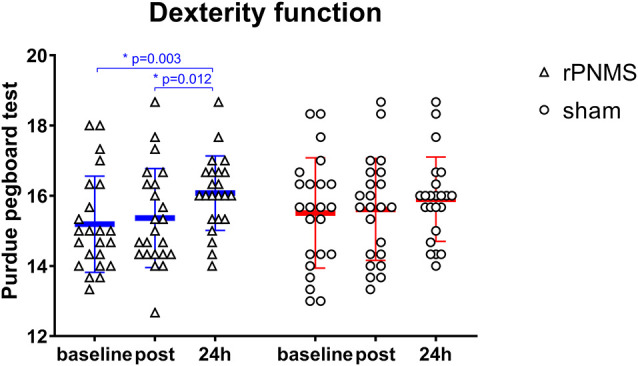
The hand dexterity function in the Purdue pegboard test was assessed at baseline, immediately after the intervention, and 24 h after interventions: rPNMS (blue line) and sham (red line). The open triangles and circles represent the individual data in the rPNMS and sham group respectively. There was no significant difference in hand dexterity change between the two groups. *Post hoc* pairwise comparison with Bonferroni correction revealed that the improvement in Purdue pegboard score was significant at 24 h afterward compared with baseline (*p = 0.003) and immediately after rPNMS (*p = 0.012).

Regarding the motor hand function, the dexterity of the upper extremity evaluated using the Purdue pegboard was significantly improved after two stimulations over time: two-way repeated-measures ANOVA revealed a significant effect of time (*F* = 10.081, *p* = 0.000) but no significant effects of group (*F* = 0.132, *p* = 0.719) or group * time interaction (*F* = 1.577, *p* = 0.212). The *post hoc* comparison showed the dexterity of the upper extremity evaluated using the Purdue pegboard was significantly improved after rPNMS (*F* = 8.851, *p* = 0.001) but not after sham stimulation (*F* = 2.088, *p* = 0.136). For rPNMS group, the pairwise comparisons revealed that the improvement in Purdue pegboard score was significant at 24 h afterwards compared with baseline (*p* = 0.003) and immediately after rPNMS (*p* = 0.012; Figure 6). There were no significant effects of time (*F* = 1.014, *p* = 0.344), group (*F* = 0.040, *p* = 0.842) or group*time interaction (*F* = 0.168, *p* = 0.765) regarding grip strength.

**Figure 7:**
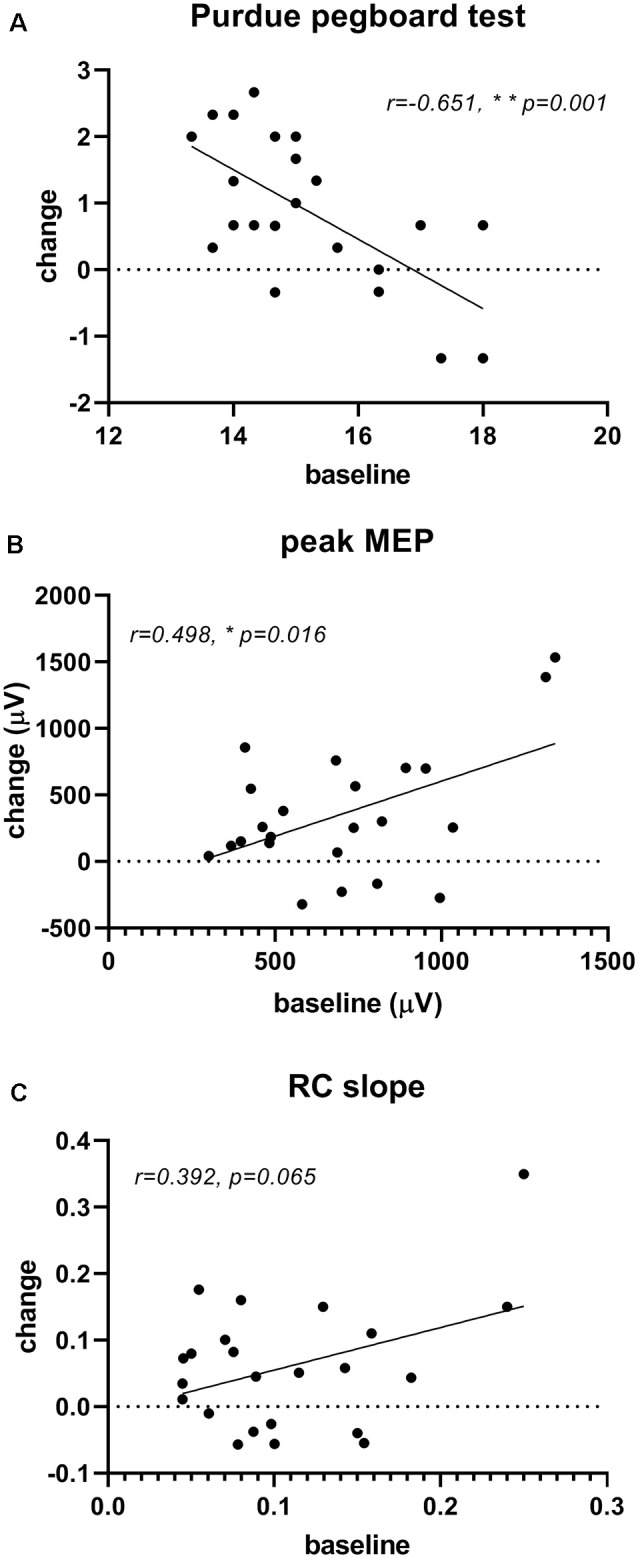
Correlation of significant changes in outcome measures after rPNMS with their baseline value: Purdue pegboard score **(A)**, peak MEP amplitude (μV; **B**) and RC slope **(C)**. The significant correlation was found for Purdue pegboard score (*r* = −0.651, *p* = 0.001) and peak MEP amplitude (*r* = 0.498, *p* = 0.016) but not for RC slope (*r* = 0.392, *p* = 0.065).

The correlation analysis demonstrated that the improvement in Purdue pegboard score (0.88 ± 1.10) and peak MEP amplitude (357.08 ± 478.99 μV) after rPNMS were negatively and positively correlated with their baseline value respectively (*r* = −0.651, *p* = 0.001 and *r* = 0.498, *p* = 0.016; Figures 7A,B), whereas the correlation of the change in RC slope with its baseline value was not significant (*r* = 0.392, *p* = 0.065; Figure 7C; Table 2).

**Table 2:** Correlation between changes in outcome measures after repetitive peripheral nerve magnetic stimulation (rPNMS) and their baseline value.

Outcome measures	Baseline (mean ± SD)	Changes (mean ± SD)	Pearson’s *r*	*p*
Purdue	15.19 ± 1.37	0.88 ± 1.10	−0.651	0.001**
RC slope	0.11 ± 0.06	0.06 ± 0.10	0.392	0.065
peak MEP	702.09 ± 288.71	357.08 ± 478.99	0.498	0.016*

*Correlation is significant and *p* < 0.05; **correlation is significant and *p* < 0.05.

## Discussion

The present study aimed to investigate the changes in corticomotor excitability and motor hand function induced by repetitive stimulation using magnetic pulses instead of conventional electrical current, and which was applied on peripheral nerves rather than on muscles as in other previous rPMS studies. This study newly showed that one session of rPNMS applied to the median nerve of the non-dominant upper extremity modestly increased the corticomotor excitability in contralateral M1 and may associate improved hand dexterity in healthy young individuals.

Gallasch et al. (2015) applied repetitive magnetic stimulation with 15,000 pulses delivered at 25 Hz for 20 min on the volar side of the right forearm to stimulate finger and wrist flexor muscles in normal subjects and showed a significant increase in the MEP amplitudes recorded at right flexors carpi radialis. Such upregulation of MEP amplitudes was associated with a decreased short-interval intracortical inhibition (SICI) and increased intracortical facilitation (ICF), which were assessed by paired-pulse TMS, as well as enhanced activation in left precentral/postcentral gyrus, as shown by fMRI scans. Similarly, the present study showed that repetitive magnetic stimulation to the median nerve of the non-dominant hand with 2,400 pulses, delivered at a slightly lower frequency of 20 Hz over 10 min, increased the RC slope and peak MEP amplitudes in the contralateral hemisphere. This observation agreed with the results of studies that demonstrated that the induction of corticomotor excitability changes after an external electrical stimulation was applied to the muscles or peripheral nerves (Charlton et al., 2003; Chipchase et al., 2011). The underlying mechanism may be that the repetitive magnetic stimulation applied on either muscle or the peripheral nerve may have induced the proprioceptive input by the direct action of the sensorimotor nerve fibers and the indirect activation of mechanoreceptors during a rhythmical contraction–relaxation and muscle vibration (Struppler et al., 2004; Momosaki et al., 2017). Such proprioceptive afferent input to the S1 along the ascending sensory pathway then might drive reorganization in M1 through the structural and functional connections between S1 and M1 (Schabrun et al., 2013). This neural circuit would play a vital role in modulatory effects of corticomotor excitability from rPNMS in the current study.

The RC represents the growth of MEP amplitudes as a function of TMS output intensity; thus, the slope reflected the neurophysiological strength of intracortical and corticospinal connections (Liu and Au-Yeung, 2014). The peak MEP during the RC assessment revealed the extent of maximal excitation caused by the recruitment of the corticospinal pathways responding to TMS (Liu and Au-Yeung, 2014). Therefore, the increase in the RC slope and peak MEP amplitudes after rPNMS in the present study might indicate the enhanced activation of corticomotor synaptic connections and the corticospinal pathways to the non-dominant APB muscle in contralateral M1. This rapid cortical plasticity was suggested to be associated with the unmasking of latent synapses and the modification of synaptic strength, which are known to be involved in the reduction of GABAergic inhibition (Chipchase et al., 2011; Beaulieu et al., 2017). The downregulation of GABAergic inhibition is the mechanism underlying peripheral electrical nerve (Kaelin-Lang et al., 2002) and magnetic muscle stimulation (Gallasch et al., 2015; Beaulieu et al., 2017). Kaelin-Lang et al. (2002) showed that the MEP amplitudes of contralateral abductor digiti minimi muscle were increased after a 2-h ulnar stimulation. However, this excitatory effect of PNS was blocked by lorazepam, which is a GABA receptor agonist. In contrast, Gallasch et al. (2015) and Beaulieu et al. (2017) demonstrated decreased SICI, which was more likely mediated by GABA-A receptors after one or multiple sessions of rPMS which were applied to stimulate the muscles in the upper limb of normal subjects and the lower limb of chronic stroke patients, respectively. Hence, it remains to be verified whether an increase in RC slope and peak MEP amplitudes after the rPNMS in this study are the after-effects of decreased GABAergic intracortical inhibition.

Gallasch et al. (2015) showed that rPMS applied to muscles could not alter the rMT in healthy subjects, whereas rMT showed non-significant changes but tended to decrease after rPNMS in the present study. As another aspect of corticomotor excitability, the motor threshold is known to depend on the voltage-gated sodium channels and reflects the membrane excitability of the corticomotor neurons in the cortical motor representation region for the target muscle (Liu and Au-Yeung, 2014; Ziemann et al., 2015). Hence, the results of the present study might indicate insufficient changes of ionotropic channels for membrane excitability enhancement of motor neurons contralateral to the APB muscle induced by a single session of rPNMS applied to the median nerve based on the current protocol and healthy sample.

Nevertheless, to take a more rigorous data analysis, the peak MEP amplitude and RC slope after rPNMS were not significantly different from their values in the sham group at baseline and immediately after stimulation even the significant group difference was found for peak MEP amplitude and statistical pre-post change for these two corticomotor excitability outcomes after rPNMS. This may indicate that the corticomotor excitability induced by peripheral magnetic stimulation applied to the median nerve with current protocol in one single session might not be robust enough. On the other hand, individual data points of peak MEP amplitude and RC slope showed two subjects had a relatively larger response for rPNMS. If these two subjects were excluded, the statistical analysis revealed non-significant group differences (time * group interaction: *F* = 2.983, *p* = 0.092) for peak MEP amplitude with the pre-post change were still significant (*p* = 0.003 for peak MEP amplitude and *p* = 0.014 for RC slope respectively). Hence, the up-regulation effects of corticomotor excitability induced by rPNMS in the present study should be treated with caution. Using repetitive sessions of stimulation, well-designed research with a larger sample and good homogeneity, and healthy subjects should be conducted attempting to elicit more robust modulatory effects of rPNMS.

Previous studies have shown that the rPMS to the muscles could increase dexterity function in stroke patients. Struppler et al. (2003, 2007) demonstrated that the velocity and amplitude of finger movements were significantly enhanced after 15 min of rPMS applied to the hand extensor muscles, and such improvement in dexterity was associated with a reduction in spasticity, which might be the main interference factor of the movements of paretic extremities in patients with spastic paresis. When rPMS is applied to the peripheral nerves, similar effects were also observed in healthy people. Using a pre-post design in normal subjects, Okudera et al. (2015) applied 600 magnetic pulses at a frequency of 20 Hz on the radial nerve of the non-dominant hand and showed that the upper limb dexterity performance was improved, which was measured with the Box and Block Test, and this improvement was sustained for at least 15 min. For hand dexterity function change in the present study, the performance of the subjects for the Purdue pegboard improved after one session of rPNMS on the median nerve of the non-dominant hand, and this improvement was exhibited as a latent effect 24 h afterward. This was in line with the results of a previous study on healthy subjects which showed that rPMS applied to the forearm flexor muscles increased the degree of elbow stabilization (Struppler et al., 2004). This augment of stabilization of the elbow joint is required and important for fine skilled movements such as grasping and manipulation during the Purdue pegboard test. Although such benefits in hand dexterity were observed after rPNMS, the between-group difference was not significant in the statistical analysis. Based on the effect size of 0.19, we deemed that the small sample size (*n* = 46) of the present study might have accounted for the non-significant difference between the two groups. To calculate with a sample size software, a sample of at least 74 (37 per group) would be required to validate the positive effects of rPNMS in improving hand dexterity function beyond that of sham intervention. On the other hand, a notable correlation was found between the amount of change of hand dexterity performance after rPNMS and their baseline values. The good performance of the Purdue pegboard test at baseline was negatively related to its improvement 24 h after rPNMS might indicate the inherent limitation of hand dexterity in healthy people. Moreover, it was noted that the baseline values of the Purdue pegboard score were slightly higher in the sham group (15.50 ± 1.57) than in the rPNMS group (15.19 ± 1.37) even the difference was not significant. Further analysis showed that the Purdue pegboard score of subjects with lower baseline values (<15.50, *n* = 11) in the sham group was also increased at 24 h after the sham stimulation (15.15 ± 0.81) compared with baseline (14.12 ± 0.82; *p* = 0.003) and immediately after sham stimulation (14.42 ± 0.87; *p* = 0.024). This could be explained that the relative inflexible upper extremity in healthy subjects might present better responsiveness to the process of the Purdue pegboard test which *per se* can be seen as a practice. However, this might raise the question of whether the improvement in hand dexterity after rPNMS was attributed to the magnetic nerve stimulation or was due to the lower baseline itself. To address this, a future study with a larger sample would show a more comparable baseline of motor hand function for different groups and should be able to respond to this argument.

A few studies have demonstrated that using multiple sessions of rPMS to stimulate limb muscles could enhance muscle strength. With the coil placed over the anterior aspect of the thigh, Yang et al. (2017) showed that both isometric and isokinetic maximum/average peak torque of quadriceps were increased significantly after 15 min of rPMS applied three times per week for five consecutive weeks, while the quadriceps strength was not changed in the control group, which performed normal activities of daily living during the 5 weeks. Musarò et al. (2019) applied the 10 sessions of daily magnetic stimulation to the forearm flexor muscles in patients with amyotrophic lateral sclerosis and showed significant improvement in the MRC-score of the flexor carpi radialis muscle and muscle strength, which was measured using a handgrip dynamometer. By contrast, no significant improvement in muscle strength was observed in the other untreated muscles and in the opposite arm which received sham stimulation. Besides, rPMS applied to certain muscles for 15–24 sessions in a period of 3–8 weeks also enhanced the muscle strength for several other conditions that may lead to muscle weakness such as COPD (Bustamante et al., 2010) as well as post-operation muscle weakness (Baek et al., 2018). The lack of improvement in handgrip strength after rPNMS on the median nerve in the present study might be due to the minimum dose administered in one single session.

Although rPNMS did not show the superior effects in motor hand function to sham stimulation in the present study, the up-regulation of corticomotor excitability, even the effects were modest, would allow it to serve as a primer delivered ahead of other interventions and may bring the possible benefits for function enhancement in clinical rehabilitation. On the other hand, the rPNMS-induced increase in peak MEP amplitude was positively related to its value before stimulation might reveal that the people with less exciting motor cortex could be more responsive to rPNMS. Hence, peak MEP amplitude may be used as a TMS outcome to predict the cortical effects when rPNMS is delivered to priming the motor cortex in clinical practice, for example, the sports training of athletes and other conditioning patients without neural system lesion.

Note that the interpretation of the results of this study might be confined to some limitations. First, a small healthy sample was recruited so that the neuromodulation and motor effects of rPNMS would not be generalized to patients with neurological diseases such as stroke. Second, the effects of rPNMS were investigated after only one session. Whether multiple sessions applied more frequently for clinical rehabilitative intervention would lead to more pronounced effects is unknown. Moreover, the corticomotor effects of rPNMS might involve the faciliatory and inhibitory modulation from the intracortical neurons. Single-pulse TMS adopted in the present study could not reveal such neurophysiological processes. Therefore, further studies using paired-pulse TMS and fMRI can examine if repeated rPNMS can induce up-regulation of corticomotor excitability and can enhance motor function as well as the associated neurophysiological processes in a larger sample of healthy subjects and heterogeneous patient populations.

To conclude, one single session of rPNMS applied to the median nerve may increase the corticomotor excitability in contralateral M1, together with a possible improvement in dexterity function of the stimulated upper extremity in healthy people. This shows that rPNMS may be applied as an intervention method for clinical rehabilitation.

## Data Availability Statement

The original contributions presented in the study are included in the article, further inquiries can be directed to the corresponding author/s.

## Ethics Statement

The studies involving human participants were reviewed and approved by Research Ethics Committee of the JORU Rehabilitation Hospital. The patients/participants provided their written informed consent to participate in this study.

## Author Contributions

YJ: study concept, subject recruitment, acquisition of data, and writing the first draft. XL: subject recruitment, acquisition of data, and data analysis. JW, DL, and CW: subject recruitment and acquisition of data. XW: experimental design, data analysis, and comments on the manuscript. HL: study concept and experimental design, data analysis and interpretation, critical revision of the manuscript, and research funding. All authors contributed to the article and approved the submitted version.

## Conflict of Interest

The authors declare that the research was conducted in the absence of any commercial or financial relationships that could be construed as a potential conflict of interest.
